# Supplementary figures and images for: The evolutionary and molecular history of a chikungunya virus outbreak lineage
Source: PLoS Negl Trop Dis. 2024 Jul 26;18(7):e0012349. doi: 10.1371/journal.pntd.0012349 (PMC11305590; doi:10.1371/journal.pntd.0012349)

# Supplementary Figure S1

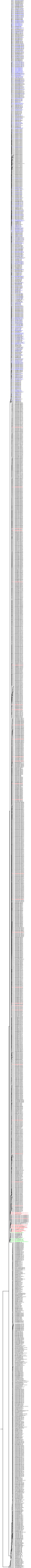

Supplement: S1 Fig — Sequences that were included in the subsampled dataset were colour coded according colours in Fig 1. (PDF) [file pntd.0012349.s001.pdf]

Supplementary Figure S2

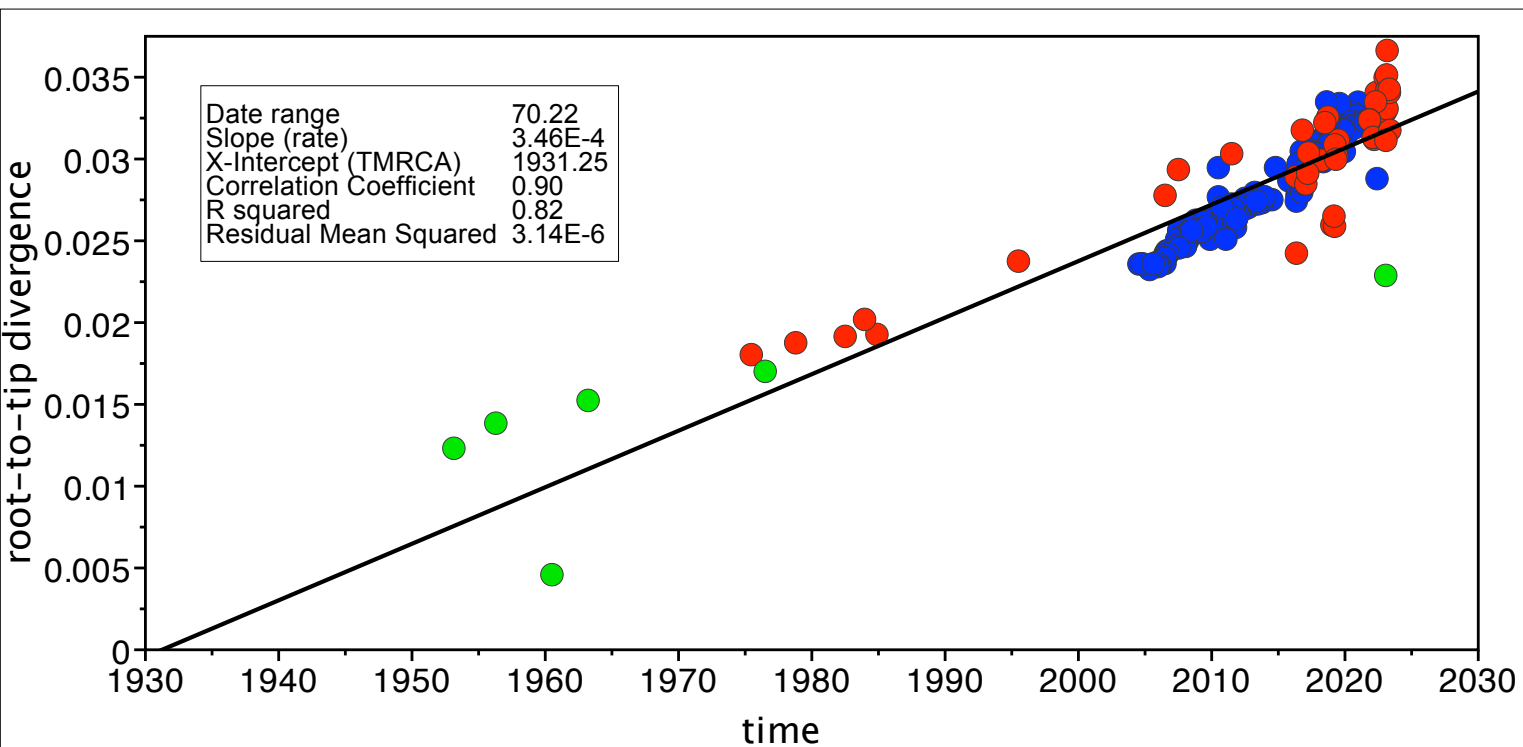

Supplement: S2 Fig — Data points were colour coded according colours in Fig 1. (PDF) [file pntd.0012349.s002.pdf]

## Supplementary Figure S3

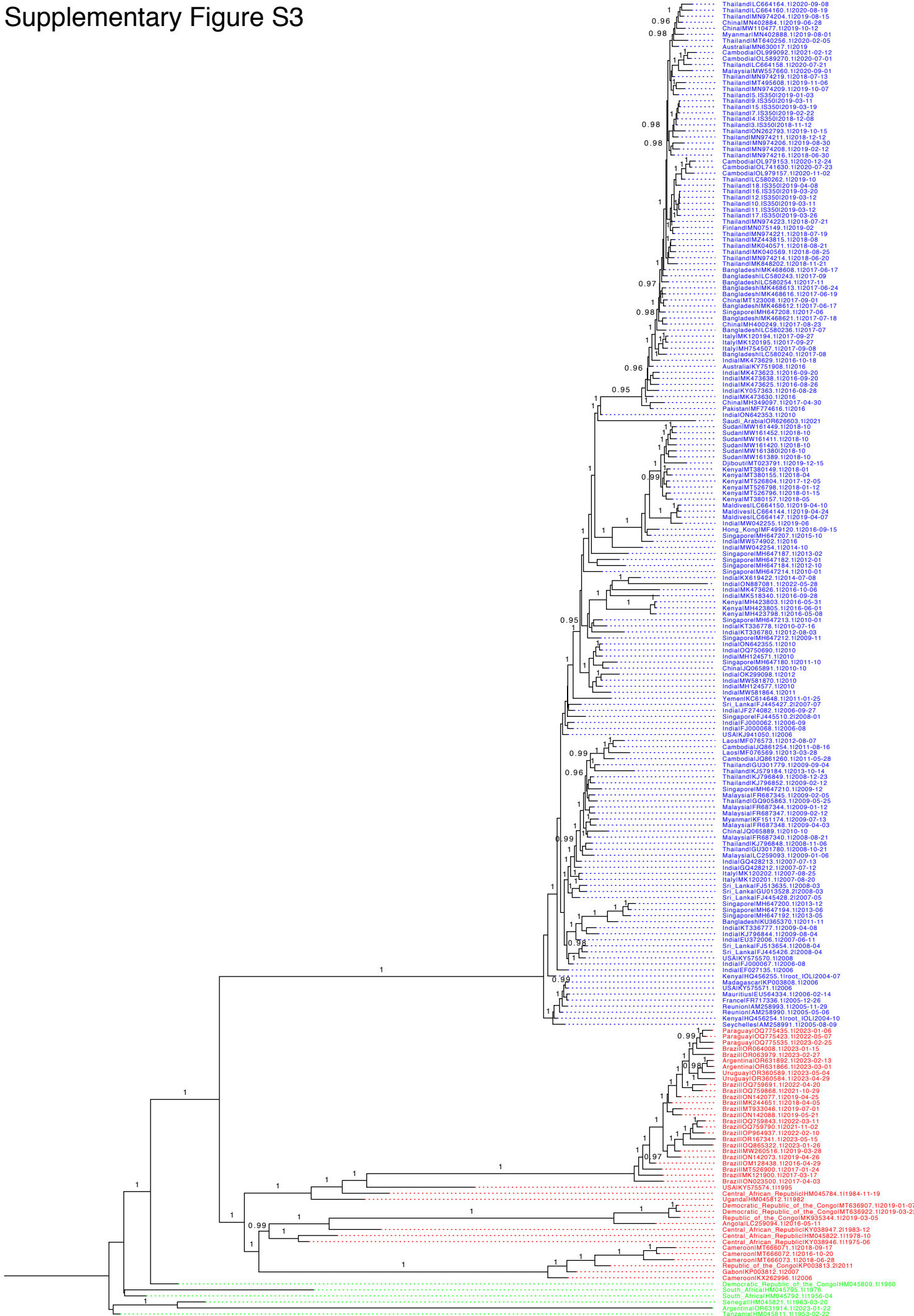

Supplement: S3 Fig — (PDF) [file pntd.0012349.s003.pdf]
