# Supplementary material for: The evolutionary and molecular history of a chikungunya virus outbreak lineage
Source: PLoS Negl Trop Dis. 2024 Jul 26;18(7):e0012349. doi: 10.1371/journal.pntd.0012349 (PMC11305590; doi:10.1371/journal.pntd.0012349)
Supplement: Attachment — Submitted filename: Response to reviewers_PNTD-D-24-00464_20240614.pdf [file pntd.0012349.s005.pdf]

Re: PLOS Neglected Tropical Diseases submission (PNTD-D-24-00464) – The evolutionary and molecular history of a chikungunya virus outbreak lineage

Point-by-point Response to reviewers

We thank the PLOS Neglected Tropical Diseases editors for considering our manuscript and for giving us the opportunity to resubmit a revised version of our manuscript. We have therefore thoroughly revised the former manuscript to account for the comments made by the reviewers. Below is our point-by-point response to each of the comments and issues raised.

Reviewer's Responses to Questions

Key Review Criteria Required for Acceptance?
Methods
-Are the objectives of the study clearly articulated with a clear testable hypothesis stated?
-Is the study design appropriate to address the stated objectives?
-Is the population clearly described and appropriate for the hypothesis being tested?
-Is the sample size sufficient to ensure adequate power to address the hypothesis being tested?
-Were correct statistical analysis used to support conclusions?
-Are there concerns about ethical or regulatory requirements being met?

Reviewer #1: The study is designed for multi-year phylogenetic analysis of CHIKV using available public data The authors sequenced 12 additional CHIKV sequence from travelers returning from Thailand. The number of samples used for the analysis is sufficient. Conclusions are very well supported by the analysis and statistical analysis employed.

Reviewer #2: yes

Authors' response:
We thank both reviewers for the time taken to review and provide constructive feedback to our manuscript. We have tried our best to address all concerns and comments raised. Please see below a response to all comments and the corresponding change in the manuscript.

Results
-Does the analysis presented match the analysis plan?
-Are the results clearly and completely presented?
-Are the figures (Tables, Images) of sufficient quality for clarity?

Reviewer #1: The analysis matches with the plan to describe the evolutionary of CHIKV outbreak. Figures and results are also not that clear (see below).

Reviewer #2: yes

Authors' response:
Thank you for the comments. Please see specific comment below regarding the figures and changes made.

Conclusions
-Are the conclusions supported by the data presented?
-Are the limitations of analysis clearly described?
-Do the authors discuss how these data can be helpful to advance our understanding of the topic under study?
-Is public health relevance addressed?

Reviewer #1: Conclusions seem to be supported by the data. The authors, however, did not mention any limitations of the analysis.

Reviewer #2: yes

Authors' response:
We agree that the limitations of our study are not made clear enough. We have therefore added more text on this in the materials and methods section of the evolutionary and mutational analyses sections to highlight limitations.

Reviewer #1: There are some minor revisions to improve the readability.
1. Please write in more detail how the sequences were assembled.

Authors' response:
We have attempted to be more specific regarding the sequence assembly and have updated this part to read:

"First, low-quality reads were removed with Trimmomatic v.0.36 [33] employing the default settings (i.e., 'ILLUMINACLIP:TruSeq3-PE.fa:2:30:10:2:True LEADING:3 TRAILING:3 MINLEN:36'). All quality-checked sequence data libraries were then mapped with Bowtie v.2.3.4. using the default local settings (i.e., --sensitive-local, same as: -D 15 -R 2 -N 0 -L 20 -i S,1,0.75) against a NCBI CHIKV reference sequence (NCBI GenBank accession number: MF773566), whereupon 50% majority consensus sequences were generated."

2. Please separate Figure 1 into Figure 1A for the phylogenetic tree and 1B for the map of the movement.

Authors' response:
We understand your suggestion to separate the figures into two. However, we believe that keeping the figures together better preserves the context and connection between the data. To enhance clarity and facilitate reference in the text, we have divided the figure into parts A and B. We hope this approach addresses your concerns while maintaining the integrity of the information.

3. There seems to be detachment of the map of the virus movement and its explanation in the manuscript. For example, line 212 says, "islands (Madagascar, Mauritius, Mayotte, Comoros) in the 1960s and to India in the 1980s (blue)", however the map shows that the year of the spread to India is 2003. Also, line 210 says, "Central Africa to the Americas (red) in the late 1980s and early 2010s (1995 in the USA and 2014", however the map shows that the spread to the USA in 1979 and to Brazil in 2013.

Authors' response:
Thank you for pointing this out. The map here shows the correct year during which the movement occurred. The correct sentence for line 212 has been corrected to:
"…islands (Madagascar, Mauritius, Mayotte, Comoros) in the 1960s and to India in the early 21st century (blue)."
We also agree that the sentence on line 210 is confusing. This stems from the fact that we could not verify that the sequence "USA|KY575574.1|1995" is a mosquito collected in the US or a human infected in the US, but rather a person infected in an African country (i.e. an imported case to the US). We have tried to clear up the description and made sure that the date in the map corresponds to what is written in the text.

4. The map in Figure 1 shows red arrow pointing from Cameroon to Madagascar. Is it not blue arrow? Also, the arrow pointing the movement of ECSA2 to ECSA1 from Uganda to Senegal in 1963 does not seem to fit the phylogenetic tree.

Authors' response:
The introduction from Cameroon to Madagascar, identified as the switch from ECSA2 to IOL, precedes the first occurrence of a sequence identified as IOL. Since first IOL occurrence, outbreak, and spread was documented in the Indian ocean islands, and arrow in question shows the origins to the Cameroonian ancestor, we chose to depict this in red to highlight the ancestor origine. Since no IOL strains have been detected to circulate in Cameroon and surroundings, it is likely that the mutations dividing the ancestral strain and the new IOL strains occurred either on the way from the continent to the islands or on the islands. Hence the colour choice.
The green arrow, pointing to Senegal in 1963, should originate from Tanzania. We acknowledge that it previously appeared as if the arrow originated in Uganda, and this has now been adjusted for clarity.

Reviewer #2: Abstract:
1) "…we also identified amino acid substitutions that may be associated 14 with immune evasion, increased spread, and virulence." and the next sentence "…which are highly relevant as they may 16 lead to changes in vector competence, transmission efficiency and pathogenicity of the virus" are duplicates. Rephrase, please.

Authors' response:
Thank you for pointing this out. This has now been changed.

2) I would prefer to have both latin names spelled full at the first encountering in the text: Aedes aegypti and Aedes albopictus (vs. Ae. albopictus) line 38

Authors' response:
This has been corrected.

3) A badly phrased sentence – line 42-43

Authors' response:
Agreed. The sentence has been modified from:
"CHIKV was first discovered in Tanzania in 1952 and has since its discovery both been identified and/or suggested to be the causative agent of multiple outbreaks in Africa and Asia for several decades, if not centuries [6, 7]."

To:

"Since the discovery of CHIKV in Tanzania in 1952, the virus has been identified as the causative agent of multiple outbreaks occurring globally and suspected to be the pathogen of outbreaks dating back centuries [6, 7]."

4) Line 91 – "mutational outbreak lineage analysis" sounds strange to me, but I am not an epidemiologists.

Authors' response:
Agreed. We have simply removed "mutational" from the sentence as "outbreak lineage analysis" is descriptive enough and that what is included in the outbreak analysis is included in the sentence directly after.

5) Line 92 – time estimation for what? Please, specify better.

Authors' response:
This has been modified to make clear that we refer to the evolutionary and geographic history of CHIKV. We have changed this to "We reconstructed the evolutionary and geographic history, divergence times and performed mutational profiling".

Methods:
1) 12 positive patients out of? I am rather curious how relevant is the study in Europe.

Authors' response:
According to the ECDC report "*Chikungunya virus disease Annual Epidemiological Report for 2019*", Sweden had 20 CHIKV cases 2018 and 58 CHIKV cases 2019. For that period (2018–2019) the cases in Sweden represented 11% of all cases registered in Europe (20 + 58 / 170 + 516), of which we sequenced 15% of the Swedish cases.

2) Line 114-115 – genomes generated above? Do author mean the consensus sequences? It can be explained a bit clearer.

Authors' response:
Correct, the line has now been changed to read "…together with the 12 CHIKV consensus genome sequences generated above…".

3) Line 123-124 – where the older datasets were taken from? Ref to the database or a source should be provided (years 1953-2023)

Authors' response:
Yes, this should be made clearer. We have added this information directly in the first sentence (line 114–116), as it applies to all datasets used, which now reads:

"First, 2,564 CHIKV sequences of ≥8000bp, with known collection date and geographic location were retrieved from NCBI GenBank and aligned together with the 12 CHIKV consensus genome sequences generated above using MAFFT v.7.520 [34, 35] utilizing the L-INS-i algorithm, where the 5' and 3' ends were trimmed."

4) Line 137 – how the mutations were mapped on the models?

Authors' response:
We revised the first sentence of "Mutational analysis" subsection to clarify the text and the steps performed.

Main text:
1) Line 235, line 268 – typos in the sentences?

Authors' response:
Thank you for noticing this, we have made the following changes:
235: "Comoros with a population of approximately 316,600, were infected with CHIKV ~~on~~"
268: "To rationalise the impact of the mutations on viral proteins function ~~of~~,"

Supplementary:

I suggest to make the big tree of the Sup. Fig.1 circular. When rectangular, it is not possible to read.

Authors' response:
We appreciate the suggestion to change the phylogenetic tree to a circular format to improve visibility. While we agree that large trees can be challenging to view in a rectangular format, we have found that the circular format exacerbates these issues. In a circular tree, sequences become harder to distinguish, and the names often overlap, rendering the tree unreadable. Although the rectangular format is not perfect, we believe it is more practical and accessible than the circular alternative. We hope you understand our decision to maintain the rectangular format for clarity.

Summary and General Comments
Reviewer #1: The manuscript by Krambich J et al. titled The Evolutionary and Molecular History of a Chikungunya Virus Outbreak Lineage is interesting for general reader. Using sequence of the CHIKV from travelers to Thailand, they are able to make a connection how the virus lineage is connected to the previous outbreaks and also able to give the reader a picture of the spread of the virus, especially the ECSA genotype. Therefore, this is a very nice paper and is good for publication after addressing the minor revisions.

Authors' response:
We sincerely thank Reviewer #1 for the valuable comments, which have significantly enhanced and clarified our manuscript.

Reviewer #2: The authors did a detailed work on tracing the spread history of the chikungunya virus around the globe. This is an important aspect of epidemiology and virology and the study helps our understanding of these processes for CHIKV and for other viruses, in general. Moreover, the analyses of the mutations and their possible consequences on the virus life cycle are a meticulous and well done work. This is also a great contribution to molecular biology and evolution of the mechanisms of viral infections. I recommend this work to publishing with some minor changes. See below.

Authors' response:
We also thank Reviewer #2 for highlighting areas of weakness in our manuscript. Their comments have greatly improved its readability and rigor.
